# Survival Impact of Bevacizumab According to Chemotherapy Response Score in Advanced High-Grade Serous Ovarian Carcinoma Treated with Neoadjuvant Chemotherapy

**DOI:** 10.3390/cancers18183045

**Published:** 2026-09-19

**Authors:** Hasan Volkan Ege, Haticegul Tuncer, Utku Akgör, Murat Gultekin, Zafer Selcuk Tuncer, Derman Basaran

**Affiliations:** 1 Department of Gynecologic Oncology, Etlik City Hospital, Ankara 06170, Turkey; 2 Faculty of Medicine, Department of Obstetrics and Gynecology, Hacettepe University, Ankara 06420, Turkey; 3 Faculty of Medicine, Department of Obstetrics and Gynecology, Division of Gynaecological Oncology, Hacettepe University, Ankara 06420, Turkey

**Keywords:** bevacizumab, chemotherapy response score, ovarian cancer, recurrent disease

## Abstract

Introduction: Chemotherapy response score (CRS) is a prognostic marker in high-grade serous ovarian carcinoma treated with neoadjuvant chemotherapy (NACT). This study evaluated whether the survival benefit associated with sustained bevacizumab exposure differs according to CRS group. Methods: We retrospectively evaluated patients who underwent interval debulking surgery after platinum-taxane-based NACT between January 2015 and February 2025. Sustained bevacizumab exposure was defined as receipt of more than three cycles. Overall survival (OS) and OS after first recurrence (OS-FR) were assessed using Kaplan–Meier and Cox regression analyses. Results: The cohort included 113 patients: 30 with sustained bevacizumab exposure and 83 in the comparison group; 74 had CRS 1–2 and 39 had CRS 3. Sustained bevacizumab was associated with longer median OS in the overall cohort (57.4 vs. 50.3 months; *p* = 0.021) and in patients with CRS 1–2 (57.1 vs. 43.6 months; *p* = 0.045), but not in those with CRS 3 (*p* = 0.224). OS-FR was also longer with sustained bevacizumab overall (35.0 vs. 23.0 months; *p* = 0.013) and in CRS 1–2 (35.0 vs. 23.0 months; *p* = 0.041). In multivariable analysis, sustained bevacizumab was independently associated with improved OS-FR (landmark-corrected HR 0.23, 95% CI 0.07–0.75; *p* = 0.015). Conclusions: Sustained bevacizumab exposure was associated with longer OS and OS-FR, particularly in patients with CRS 1–2. CRS may help identify patients most likely to benefit from bevacizumab, although these findings require prospective validation.

## Simple Summary

Chemotherapy response score (CRS) reflects how well a tumor responds to chemotherapy given before surgery in advanced high-grade serous ovarian carcinoma, and bevacizumab is frequently added to treatment to improve outcomes. The aim of our retrospective study was to assess whether the survival benefit of bevacizumab differs according to CRS group. In a cohort of 113 patients, we confirmed that sustained bevacizumab exposure (>3 cycles) was associated with longer median overall survival (57.4 vs. 50.3 months) and median survival after recurrence (35.0 vs. 23.0 months) compared with limited exposure. This benefit was concentrated among the patients with CRS 1–2 (suboptimal response), while the patients with CRS 3 (near-complete response) showed no significant survival advantage. Bevacizumab exposure remained an independent predictor of improved survival after recurrence on multivariable analysis. CRS may therefore serve as a practical biomarker for selecting patients most likely to benefit from bevacizumab.

## 1. Introduction

Ovarian cancer is one of the most significant gynecologic malignancies, associated with substantial morbidity and mortality in women. According to the most recent GLOBOCAN estimates, ovarian cancer accounted for approximately 330,000 new cases and 203,000 deaths worldwide in 2024, and continues to carry one of the highest mortality-to-incidence ratios among solid tumors [1]. The disease is most commonly diagnosed at an advanced stage, namely FIGO stage III–IV [2]. The standard treatment approach for advanced epithelial ovarian cancer consists of a combination of cytoreductive surgery and platinum-taxane–based chemotherapy [3]. Primary debulking surgery (PDS) followed by adjuvant chemotherapy is the preferred approach when complete macroscopic resection is feasible and the patient is fit for surgery. In contrast, neoadjuvant chemotherapy (NACT) followed by interval debulking surgery (IDS) is employed in patients in whom complete cytoreduction is not anticipated with upfront surgery or who are not medically fit for primary surgery [4]. The primary aim of NACT is to reduce tumor burden, thereby increasing the likelihood of optimal or complete cytoreduction and reducing surgical morbidity [4]. Several randomized controlled trials have demonstrated that NACT followed by IDS is non-inferior to primary cytoreductive surgery in selected patients with advanced ovarian cancer [5,6]. In line with these results, the frequency of IDS after NACT in advanced-stage ovarian cancer has been steadily increasing [7].

The chemotherapy response score (CRS) is a standardized histopathological scoring system developed to assess the degree of response to NACT in patients with high-grade serous tubo-ovarian carcinoma [8]. Based primarily on evaluation of omental tumor regression, patients are classified into three groups: CRS 3, indicating complete or near-complete response; CRS 2, indicating substantial but incomplete response; and CRS 1, indicating no or minimal response [9]. More specifically, CRS 1 denotes no or minimal tumor response, with mainly viable tumor and no or minimal regression-associated fibroinflammatory change limited to a few foci; CRS 2 reflects an appreciable tumor response, with readily identifiable viable tumor ranging from multifocal or diffuse regression-associated change with tumor present in sheets, streaks, or nodules, to extensive regression with multifocal but easily identifiable residual tumor; and CRS 3 denotes complete or near-complete response, with no residual tumor or only minimal, irregularly scattered tumor foci (individual cells, cell groups, or nodules) up to 2 mm in maximum size [8]. The CRS has been shown to have prognostic value, particularly for progression-free survival, and has been proposed as a potential prognostic marker for overall survival in this setting [9,10]. In general, CRS 3 is associated with more favorable survival outcomes compared with CRS 1–2 [8,11]. However, there are also studies in the literature indicating that the use of CRS for predicting prognosis is limited [12].

Bevacizumab is a recombinant humanized monoclonal antibody targeting vascular endothelial growth factor-A (VEGF-A) and represents one of the targeted therapeutic agents approved for use in combination with chemotherapy in both newly diagnosed advanced ovarian cancer and recurrent disease [2,13,14,15]. The most consistent effect of bevacizumab in ovarian cancer has been improvement in progression-free survival [16,17]. Its impact on overall survival (OS), however, has been more variable and has been suggested to be more pronounced in high-risk patient subgroups [18,19,20]. In addition to contributing to survival, bevacizumab has been shown to improve treatment response rates [17]. In the recurrent disease setting, bevacizumab-containing regimens may contribute to disease control and influence survival outcomes in selected patient populations. Recent meta-analyses evaluating the addition of bevacizumab to neoadjuvant chemotherapy have reported improved rates of optimal cytoreduction but inconsistent effects on progression-free and overall survival, underscoring the need for better patient selection strategies [21,22].

Patients with CRS 1–2 following NACT represent a group with suboptimal pathological response and potentially worse prognosis. Whether the survival impact of bevacizumab differs according to CRS group therefore has important clinical implications. However, data specifically evaluating the survival effect of sustained bevacizumab use according to CRS in patients with high-grade serous ovarian carcinoma undergoing NACT and IDS remain limited. Specifically, it remains unclear whether the histopathological response captured by CRS can help identify which patients are most likely to derive a survival benefit from sustained bevacizumab exposure, a gap this study aims to address. This study aimed to evaluate the association between sustained bevacizumab exposure and OS and overall survival after first recurrence (OS-FR) according to CRS group in patients with advanced high-grade serous ovarian carcinoma treated with NACT and IDS.

## 2. Methods

This retrospective single-center cohort study included patients treated for advanced ovarian cancer at our institution between January 2015 and February 2025 who underwent IDS following NACT. Data were obtained from hospital records, operative notes, pathology reports, and follow-up documentation.

Eligible patients had received at least three cycles of platinum-taxane–based NACT, subsequently underwent IDS, survived at least three months following IDS, and had an evaluable CRS. CRS 1 and CRS 2 patients were combined into a single CRS 1–2 group given the limited number of CRS 1 cases and their shared representation of suboptimal pathological response. Bevacizumab was administered as part of adjuvant chemotherapy regimens following IDS and/or during treatment for recurrent disease. As this was a retrospective, real-world cohort spanning a decade, bevacizumab was not administered according to a standardized institutional protocol; the decision to initiate, continue, or discontinue bevacizumab was made by the treating multidisciplinary oncology board on a combination of factors including disease burden and stage, performance status, toxicity, patient preference, and evolving national reimbursement criteria over the study period. Patients who received more than three cycles of bevacizumab across their entire treatment course were classified as having sustained bevacizumab exposure; those who received three or fewer cycles or none constituted the comparison group. Because exposure status was determined from the cumulative number of cycles received over the entire treatment course, this definition is inherently susceptible to immortal time bias, as patients had to survive long enough to receive additional cycles before being classified as sustained users. OS was calculated from the date of IDS to death or last follow-up. Because bevacizumab was initiated at heterogeneous time points relative to IDS (adjuvant versus recurrent setting), a uniform bias-corrected reanalysis of OS was not feasible; this limitation is discussed below. OS-FR was defined as the interval from first recurrence or progression to death or last follow-up, and was analyzed exclusively in patients who developed recurrence. The diagnosis of recurrence or progression was established by a multidisciplinary tumor board in accordance with standard clinical practice, based on a combination of radiological findings (using RECIST 1.1 criteria where appropriate), rising CA-125 levels, and/or clinical symptoms. To mitigate immortal time bias for OS-FR, a landmark sensitivity analysis was additionally performed. The landmark time was set at 9 weeks after recurrence, corresponding to the interval required to complete four cycles of bevacizumab at the standard 3-weekly schedule used at our institution. Patients who died or were censored before reaching this landmark were excluded from the landmark analysis; among the remaining patients, bevacizumab exposure was classified according to cumulative cycles received, and survival time was recalculated from the landmark date.

Survival analyses were performed using the Kaplan–Meier method. The Breslow (generalized Wilcoxon) test was pre-specified for Kaplan–Meier comparisons given the pattern of early survival curve separation. Unlike the log-rank test, the Breslow test weights differences between survival curves by the number of patients at risk at each event time, giving proportionally greater weight to early events; this was considered more appropriate given the early divergence observed between exposure groups. Univariable and multivariable Cox proportional hazards regression analyses were performed for OS and OS-FR. The multivariable model included sustained bevacizumab exposure, CRS group, age, FIGO stage, and log-transformed CA-125. An interaction term between CRS group and sustained bevacizumab was incorporated to test for differential bevacizumab effect by CRS group. Categorical variables were compared using the chi-square or Fisher’s exact test; continuous variables using the independent samples *t*-test. A two-sided *p* < 0.05 was considered statistically significant. All analyses were performed using IBM SPSS Statistics version 26.0 (IBM Corp., Armonk, NY, USA).

## 3. Results

**Figure 1:**
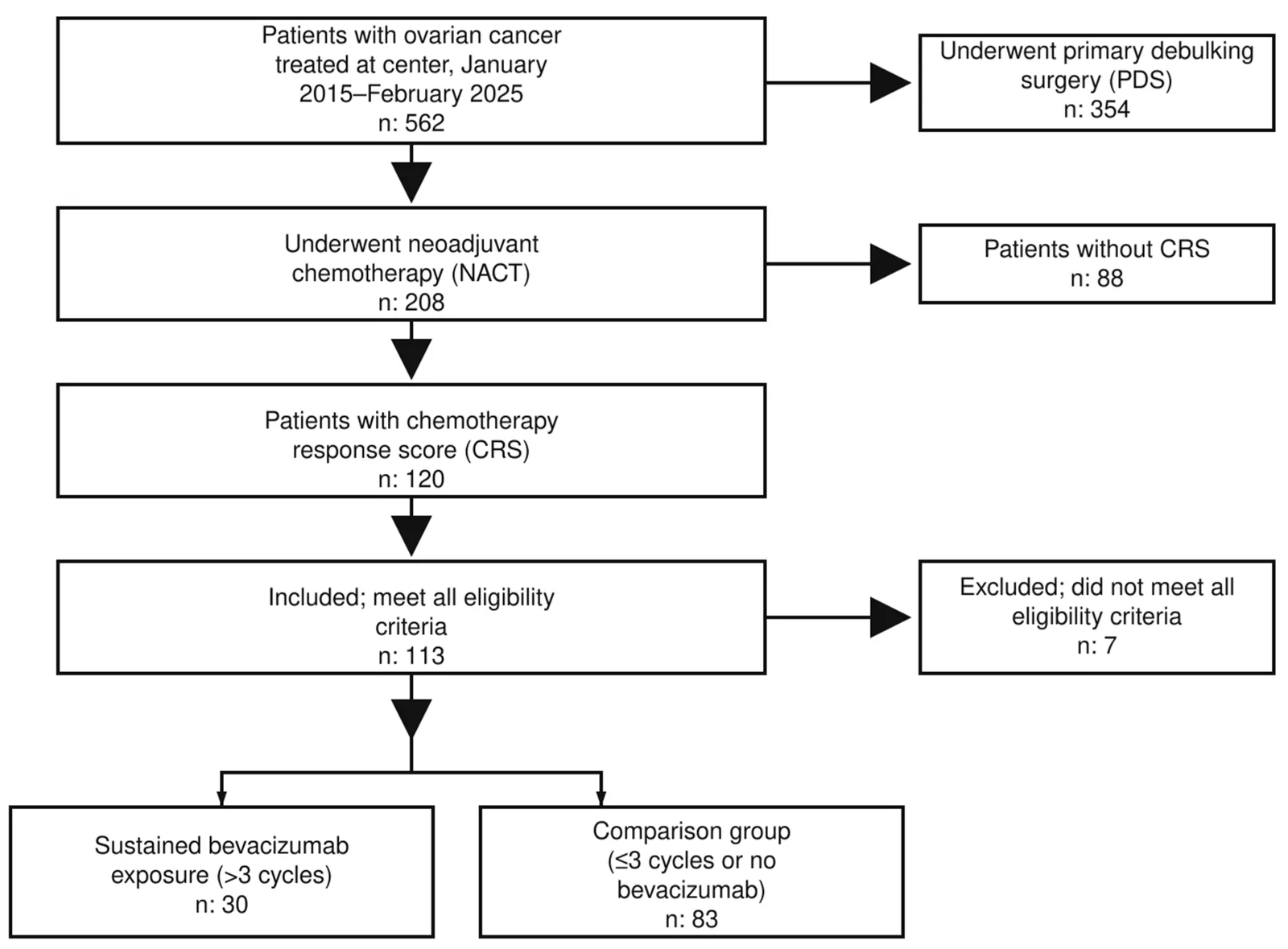
Patient flow diagram showing the selection of the final analytic cohort from all patients with ovarian cancer treated at the institution during the study period. *Abbreviations: NACT, neoadjuvant chemotherapy; IDS, interval debulking surgery; PDS, primary debulking surgery; CRS, chemotherapy response score.*

During the study period (January 2015–February 2025), a total of 562 patients with ovarian cancer were treated at our institution (Figure 1). Of these, 208 patients (37.0%) received NACT, among whom 95.1% had high-grade serous histology. After applying the eligibility criteria described above, the final analytic cohort comprised 113 patients: 30 in the sustained bevacizumab group (>3 cycles) and 83 in the comparison group. CRS distribution was: CRS 1 in 15 patients (13.0%), CRS 2 in 59 (51.3%), and CRS 3 in 39 (33.9%), yielding 74 patients in the CRS 1–2 group and 39 in the CRS 3 group. Baseline characteristics are presented in Table 1. All variables were well balanced between bevacizumab groups, including CRS distribution when classified as CRS 1–2 versus CRS 3 (73.3% vs. 62.7%; *p* = 0.406). No statistically significant differences were observed in age, BMI, laboratory parameters, CA-125, ascites, pleural effusion, FIGO stage, genetic mutation status, NACT cycles, or surgery complexity score (all *p* > 0.05). In the majority of patients (71.2%), the use of bevacizumab occurred during disease recurrence. Among the 30 patients with sustained bevacizumab exposure, 12 received it in the adjuvant setting after IDS and 20 during treatment for recurrent disease (some patients received bevacizumab in both settings), with a mean of 10.9 cycles overall; among the patients who received bevacizumab but did not meet the sustained-exposure threshold, 2 received it adjuvantly and 12 during recurrence, with a mean of 2.0 cycles. Overall, the bevacizumab-exposed population was thus predominantly composed of patients treated at the time of recurrence rather than in the adjuvant setting.

**Figure 2:**
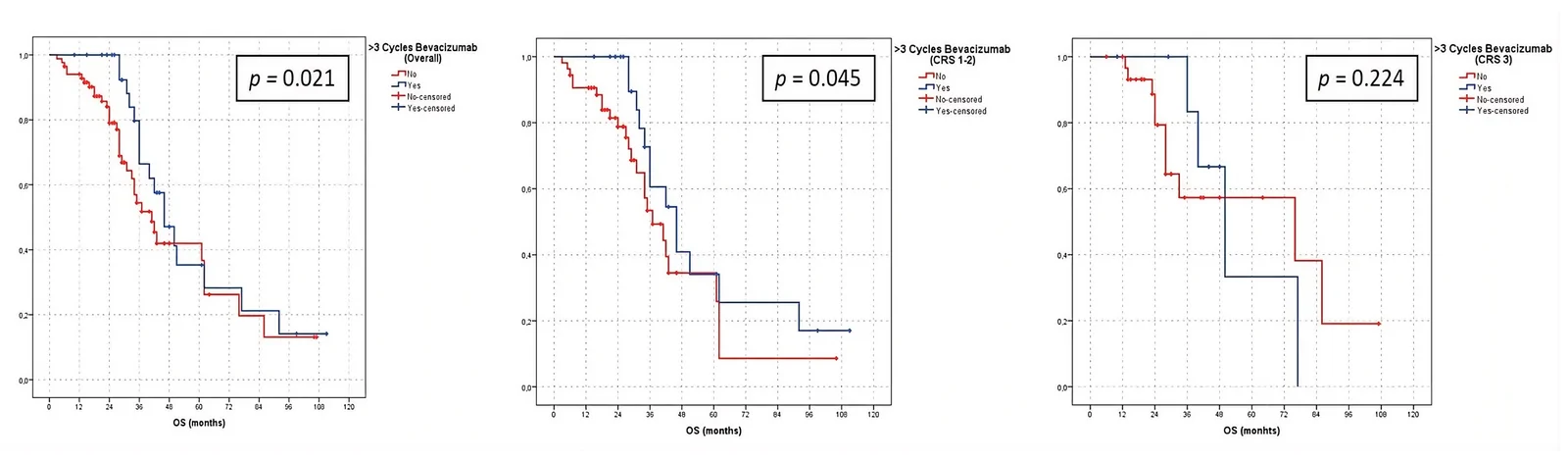
Kaplan–Meier curves for OS according to sustained bevacizumab exposure in the overall cohort (*p* = 0.021), the CRS 1–2 subgroup (*p* = 0.045), and the CRS 3 subgroup (*p* = 0.224).

**Figure 3:**
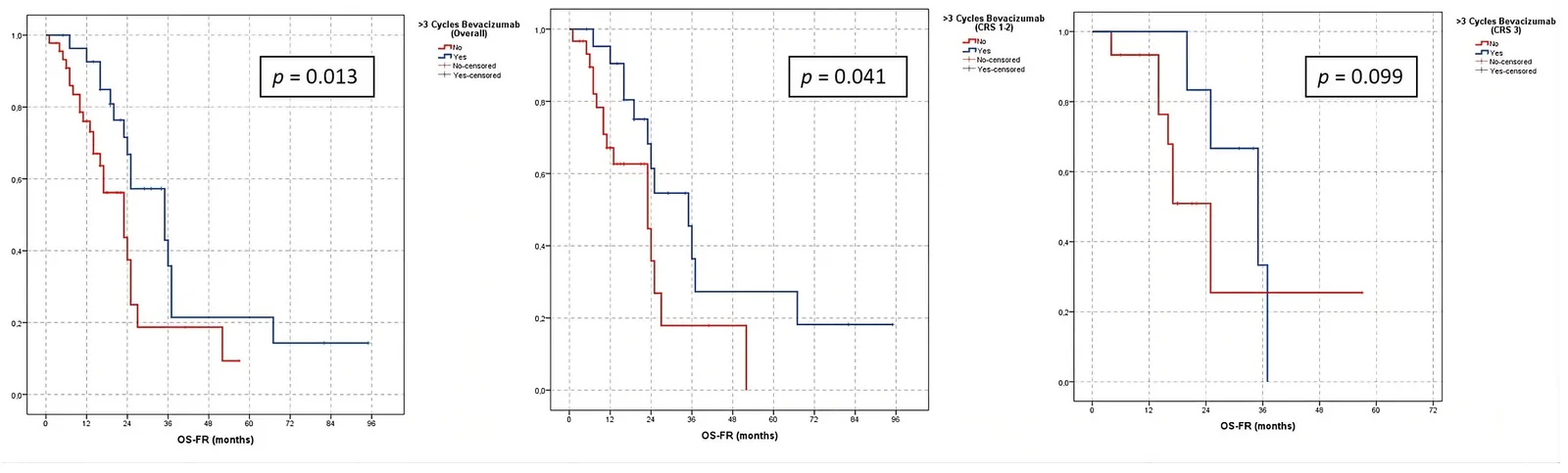
Kaplan–Meier curves for OS-FR according to sustained bevacizumab exposure in the overall recurrent cohort (*p* = 0.013), the CRS 1–2 subgroup (*p* = 0.041), and the CRS 3 subgroup (*p* = 0.099).

Kaplan–Meier survival estimates are presented in Table 2. Among patients with available follow-up data (*n* = 113), 49 deaths occurred during the study period, with a reverse Kaplan–Meier median follow-up of 41.0 months; among the 84 patients who developed recurrence, 42 deaths occurred, with a median follow-up of 24.0 months from the date of recurrence. For OS, sustained bevacizumab was associated with longer median survival in the overall cohort (57.4 vs. 50.3 months; *p* = 0.021) and in the CRS 1–2 subgroup (57.1 vs. 43.6 months; *p* = 0.045), while no significant difference was observed in the CRS 3 subgroup (55.0 vs. 62.1 months; *p* = 0.224) (Figure 2). For OS-FR, sustained bevacizumab was associated with significantly longer survival in the overall recurrent cohort (35.0 vs. 23.0 months; *p* = 0.013) and in the CRS 1–2 subgroup (35.0 vs. 23.0 months; *p* = 0.041) (Figure 3). No significant difference was observed in the CRS 3 subgroup (35.5 vs. 25.0 months; *p* = 0.099).

On multivariable Cox regression for OS (Table 3), after adjustment for CRS group, age, FIGO stage, log CA-125, and residual disease status, sustained bevacizumab was independently associated with a significantly reduced hazard of death (HR 0.27, 95% CI 0.09–0.84; *p* = 0.024). Because the model includes a CRS×bevacizumab interaction term, this hazard ratio specifically reflects the effect of sustained bevacizumab within the reference (CRS 1–2) subgroup rather than the overall cohort; refitting the model with CRS 3 as the reference category yielded a bevacizumab hazard ratio of 0.43 (95% CI 0.05–3.46; *p* = 0.430) within the CRS 3 subgroup. CRS 3 was not independently associated with OS (HR 1.17, 95% CI 0.33–4.16; *p* = 0.812). The interaction term between CRS group and sustained bevaci-zumab did not reach significance (HR 1.58, 95% CI 0.16–15.59; *p* = 0.695).

In the multivariable Cox model for OS-FR (Table 4), sustained bevacizumab was independently associated with a significantly reduced hazard of death after recurrence (landmark-corrected HR 0.23, 95% CI 0.07–0.75; *p* = 0.015). Because the model includes a CRS×bevacizumab interaction term, this hazard ratio specifically reflects the effect of sustained bevacizumab within the reference (CRS 1–2) subgroup rather than the overall cohort; refitting the model with CRS 3 as the reference category yielded a bevacizumab hazard ratio of 0.31 (95% CI 0.03–3.08; *p* = 0.319) within the CRS 3 subgroup. FIGO stage IV was independently associated with lower hazard compared with stage III (HR 0.29, 95% CI 0.12–0.67; *p* = 0.004), and higher log CA-125 with increased hazard (HR 1.38, 95% CI 1.04–1.83; *p* = 0.027). CRS group was not independently associated with OS-FR (HR 1.00, 95% CI 0.37–2.72; *p* = 0.993). The interaction between CRS group and sustained bevacizumab did not reach significance (HR 1.39, 95% CI 0.22–8.94; *p* = 0.728), a finding that warrants prospective evaluation.

## 4. Discussion

In this retrospective single-center cohort study, we evaluated the association between sustained bevacizumab exposure and survival outcomes according to CRS group in patients with advanced high-grade serous ovarian carcinoma treated with NACT and IDS. Sustained bevacizumab exposure (>3 cycles) was associated with significantly longer OS in the overall cohort and in the CRS 1–2 subgroup, while no significant benefit was observed in the CRS 3 subgroup. A similar pattern was observed for OS-FR, where sustained bevacizumab was independently associated with a significantly reduced hazard of death after recurrence (landmark-corrected HR 0.23, 95% CI 0.07–0.75; *p* = 0.015). Taken together, these findings demonstrate a consistent pattern of longer survival associated with sustained bevacizumab exposure in patients with suboptimal histopathological response (CRS 1–2). However, because the interaction between CRS group and bevacizumab exposure was not statistically significant, these subgroup findings should be interpreted as exploratory and hypothesis-generating rather than as definitive evidence of a differential treatment effect.

The impact of bevacizumab on OS in advanced ovarian cancer has been inconsistent across randomized trials. In the GOG-0218 trial, bevacizumab maintenance significantly improved PFS but failed to demonstrate a significant OS benefit in the final analysis [13]. The ICON7 trial similarly showed no OS advantage in the overall population; however, a significant benefit was observed in a prespecified high-risk subgroup, with a restricted mean survival time of 39.3 versus 34.5 months in favor of bevacizumab (HR 0.78; *p* = 0.03), suggesting that the OS benefit of bevacizumab in the first-line setting may be limited to patients with high-risk disease characteristics [18]. In the present study, similar results were observed, with sustained bevacizumab exposure associated with significantly longer OS in the CRS 1–2 subgroup—a population that shares key features with the high-risk patients who benefited most in ICON7. These findings suggest that bevacizumab should be considered more decisively in patients with suboptimal pathological response to NACT, as this group may represent those most likely to derive a meaningful survival benefit.

In the recurrent disease setting, the OS benefit of bevacizumab has similarly remained elusive in most trials. The OCEANS trial demonstrated a significant PFS improvement with the addition of bevacizumab to carboplatin-gemcitabine in platinum-sensitive recurrent ovarian cancer, yet no significant OS benefit was observed (33.6 vs. 32.9 months; HR 0.95) [23]. The AURELIA trial, conducted in platinum-resistant disease, likewise reported a PFS benefit without a significant improvement in OS [16]. The MITO16B trial, which evaluated bevacizumab beyond progression in platinum-sensitive recurrent disease, demonstrated improved PFS but again failed to show an OS advantage [24]. In contrast, the GOG-0213 trial reported a clinically meaningful improvement in median OS with the addition of bevacizumab to paclitaxel-carboplatin followed by maintenance in platinum-sensitive recurrent disease (42.2 vs. 37.3 months; HR 0.83), representing the strongest OS signal for bevacizumab in the recurrent setting to date [20]. In our cohort, the majority of patients received bevacizumab during treatment for recurrent disease, and the observed survival benefit—particularly the significantly improved OS-FR in the sustained bevacizumab group—may therefore largely reflect the impact of bevacizumab exposure in the recurrent setting. This finding is consistent with and further supports the OS signal reported in GOG-0213, reinforcing the notion that sustained bevacizumab exposure in recurrent disease may translate into a meaningful survival advantage in selected patients.

An additional finding worth noting was the observed protective association of FIGO stage IV compared with stage III in the multivariable models, particularly for OS-FR (HR 0.29, 95% CI 0.12–0.67; *p*  =  0.004), which attenuated to non-significance in the smaller, residual-disease-adjusted OS model (HR 0.66, 95% CI 0.26–1.69; *p*  =  0.388). This finding runs counter to the expected direction and should be interpreted with considerable caution. It may reflect chance, given the modest sample size and multiple comparisons involved, or possibly unmeasured selection factors specific to this single-center cohort. We do not consider this finding robust enough to draw clinical conclusions from, and it would require replication in larger, multicenter studies before any interpretation is warranted.

The differential bevacizumab benefit observed according to CRS group may reflect inherent differences in tumor biology between CRS 1–2 and CRS 3 tumors. Tumors with suboptimal pathological response to NACT likely represent a more chemoresistant phenotype with sustained angiogenic activity, potentially rendering them more susceptible to anti-angiogenic therapy. In contrast, tumors achieving a complete or near-complete pathological response are chemosensitive, with a different biological profile that limits the additive effect of bevacizumab. This interpretation is consistent with KELIM-based analyses demonstrating that patients with poor tumor chemosensitivity derive the greatest benefit from bevacizumab [19].

The multivariable analysis for OS demonstrated a significant reduction in hazard with sustained bevacizumab exposure among CRS 1–2 patients (HR 0.27, 95% CI 0.09–0.84; *p* = 0.024), though this estimate reflects the CRS 1–2–specific effect given the interaction term in the model. The apparent discrepancy between the significant Breslow-based Kaplan–Meier comparison and the non-significant univariable Cox hazard ratio likely reflects the early-weighted nature of the Breslow test relative to the Cox model, which weights the entire follow-up period equally and is therefore less sensitive to a treatment effect concentrated in the earlier part of follow-up. This finding contrasts with several large randomized trials, such as GOG-0218, which failed to demonstrate a significant OS benefit for bevacizumab in the overall population; this discrepancy should be interpreted with caution given the retrospective design and modest sample size of our cohort, and may in part reflect the additional adjustment for residual disease status in our model [13]. In contrast, the multivariable model for OS-FR identified sustained bevacizumab as an independent predictor of improved survival after recurrence (landmark-corrected HR 0.23, 95% CI 0.07–0.75; *p* = 0.015), suggesting that the survival impact of bevacizumab may be more readily detectable in the post-recurrence setting.

This study has several strengths, including its focus on the understudied question of bevacizumab benefit according to CRS group and the simultaneous analysis of OS and OS-FR. However, the retrospective single-center design, relatively small sample size, and heterogeneity in bevacizumab timing and duration are important limitations that should be considered when interpreting the findings. Importantly, defining sustained exposure using the cumulative number of cycles received over the entire treatment course introduces a risk of immortal time bias, since patients had to remain alive long enough to accrue additional cycles before being classified as sustained users. This bias could not be fully corrected for OS because bevacizumab was initiated at heterogeneous time points (adjuvant versus recurrent setting). For OS-FR, we addressed this limitation through a landmark sensitivity analysis that excluded patients who did not survive to a pre-specified landmark time after recurrence; the association between sustained bevacizumab exposure and OS-FR remained statistically significant in this bias-corrected analysis, supporting the robustness of this finding. The OS results, in contrast, should be interpreted with appropriate caution given this unresolved source of bias. An additional limitation is that bevacizumab administered in the adjuvant setting and during treatment for recurrent disease were analyzed together; granular data on treatment line, cumulative cycles by setting, and reasons for early discontinuation were not available in this retrospective cohort to allow a fully separate analysis by treatment setting. Similarly, although we incorporated residual disease status into the multivariable models (Table 3 and Table 4), data on platinum sensitivity, BRCA/HRD status, ECOG performance status, PARP inhibitor use, and subsequent lines of therapy were either incomplete or could not be reliably reconstructed for the full cohort; these unmeasured or partially measured factors may confound the observed associations. The relatively modest number of events, particularly for the OS-FR landmark analysis (24 events), limits the precision of the multivariable estimates and warrants replication in larger cohorts.

## 5. Conclusions

In conclusion, sustained bevacizumab exposure was associated with longer OS and OS-FR in patients with suboptimal pathological response to NACT, particularly in the CRS 1–2 subgroup. However, because the interaction between CRS group and bevacizumab exposure was not statistically significant, the present findings do not establish CRS as a predictive marker of bevacizumab benefit. Rather, they identify a clinically relevant survival pattern that warrants confirmation in larger prospective studies designed to evaluate whether pathological response to NACT can help refine patient selection for bevacizumab therapy. These findings should be regarded as hypothesis-generating rather than confirmatory. Future prospective studies incorporating molecular and clinicopathological correlates of angiogenic dependence alongside CRS may help define distinct risk groups more likely to derive benefit from sustained bevacizumab exposure, ultimately supporting a more individualized approach to its use in patients undergoing NACT for advanced ovarian cancer.

## Figures and Tables

**Table 1:** Baseline characteristics according to sustained bevacizumab exposure.

Variable	>3 Cycles Bevacizumab (*n* = 30)	≤3 Cycles/no Bevacizumab (*n* = 83)	*p* Value
**Continuous variables**—**mean ± SD**			
Age, years	59.9 ± 9.8	60.8 ± 9.8	0.679
BMI, kg/m^2^	30.8 ± 5.9	30.8 ± 5.9	0.975
Hemoglobin, g/dL	12.5 ± 1.2	12.1 ± 1.3	0.127
Platelet count, ×10^3^/μL	422.5 ± 155.5	409.2 ± 135.1	0.682
White blood cell count, ×10^3^/μL	8.7 ± 3.2	8.5 ± 2.9	0.732
Neutrophil-to-lymphocyte ratio	4.2 ± 2.4	4.1 ± 2.2	0.779
Albumin, g/dL	3.6 ± 0.5	3.7 ± 0.6	0.489
CA-125, U/mL	3561.6 ± 5956.9	2508.5 ± 3087.1	0.243
**Categorical variables—n (%)**			
Ascites ^a^			0.174
Yes	29 (96.7)	69 (83.1)	—
No	1 (3.3)	11 (13.3)	—
Pleural effusion ^a^			0.485
Yes	13 (43.3)	38 (45.8)	—
No	17 (56.7)	42 (50.6)	—
FIGO stage			0.620
III	14 (46.7)	45 (54.2)	—
IV	16 (53.3)	38 (45.8)	—
Chemotherapy response score			
CRS 1–2	22 (73.3)	52 (62.7)	0.406
CRS 3	8 (26.7)	31 (37.3)	—
Germline mutation status ^b^			1.000
Positive	8 (57.1)	21 (52.5)	—
Negative	6 (42.9)	19 (47.5)	—
NACT cycles			0.215
≤3 cycles	25 (83.3)	63 (75.9)	—
≥4 cycles	3 (10.0)	20 (24.1)	—

*Values are presented as mean ± SD for continuous variables and n (%) for categorical variables. p Values derived from independent samples t-test or chi-square/Fisher’s exact test as appropriate. Bold p values indicate statistical significance (p < 0.05). ^a^ The table was created by excluding missing data. ^b^ Genetic testing performed in 14/30 (46.7%) and 40/83 (48.2%) patients respectively; percentages reflect tested patients only. Abbreviations: BMI, body mass index; CA-125, cancer antigen 125; CRS, chemotherapy response score; FIGO, International Federation of Gynecology and Obstetrics; NACT, neoadjuvant chemotherapy; SD, standard deviation.*

**Table 2:** Kaplan–Meier survival estimates according to sustained bevacizumab exposure and CRS group.

Group	>3 Cycles Bevacizumab	≤3 Cycles/No Bevacizumab	*p* Value	*n*
**Overall Survival**—**median (months)**				
Overall cohort	57.4	50.3	**0.021**	113
CRS 1–2	57.1	43.6	**0.045**	74
CRS 3	55.0	62.1	0.224	39
**Overall Survival After First Recurrence (OS-FR)**—**median (months)**				
Overall recurrent cohort	35.0	23.0	**0.013**	73
CRS 1–2	35.0	23.0	**0.041**	52
CRS 3	35.5	25.0	0.099	21

*Bold p values indicate p < 0.05. p values derived from the Breslow (generalized Wilcoxon) test, as pre-specified in the Methods. OS-FR analysis restricted to patients with documented recurrence or progression. OS-FR estimates reflect a landmark-corrected sensitivity analysis (landmark: recurrence + 9 weeks) performed to address immortal time bias; see Methods. Abbreviations: CRS, chemotherapy response score; OS-FR, overall survival after first recurrence.*

**Table 3:** Multivariable Cox regression analysis for overall survival.

Variable	Univariable HR (95% CI)	*p*	Multivariable HR (95% CI)	*p*	
>3 cycles bevacizumab	0.68 (0.38–1.22)	0.195	0.27 (0.09–0.84)	**0.024**	
CRS 3 vs. CRS 1–2	0.75 (0.41–1.38)	0.356	1.17 (0.33–4.16)	0.812	
Age, per year	1.03 (0.99–1.06)	0.120	1.01 (0.97–1.06)	0.605	
FIGO stage IV vs. III	0.64 (0.35–1.16)	0.141	0.66 (0.26–1.69)	0.388	
log CA-125	1.17 (0.96–1.42)	0.122	1.33 (1.01–1.75)	**0.043**	
Residual disease present vs. absent	1.60 (0.69–3.69)	0.275	2.01 (0.75–5.35)	0.165	
>3 cycles bevacizumab × CRS 3 (interaction)	—	—	1.58 (0.16–15.59)	0.695	NS

*Bold p values indicate p < 0.05. NS = not significant. Abbreviations: HR, hazard ratio; CI, confidence interval; CRS, chemotherapy response score.*

**Table 4:** Multivariable Cox regression analysis for overall survival after first recurrence.

Variable	Univariable HR (95% CI)	*p*	Multivariable HR (95% CI)	*p*	
>3 cycles bevacizumab	0.49 (0.25–0.95)	**0.035**	0.23 (0.07–0.75)	**0.015**	
CRS 3 vs. CRS 1–2	0.92 (0.46–1.86)	0.821	1.05 (0.27–4.13)	0.941	
Age, per year	1.00 (0.97–1.04)	0.927	1.01 (0.96–1.06)	0.716	
FIGO stage IV vs. III	0.46 (0.23–0.93)	**0.031**	0.54 (0.19–1.50)	0.235	
log CA-125	1.25 (0.98–1.59)	0.069	1.22 (0.89–1.66)	0.211	
Residual disease present vs. absent	1.70 (0.72–3.98)	0.225	2.59 (0.93–7.27)	0.070	
>3 cycles bevacizumab × CRS 3 (interaction)	—	—	1.38 (0.13–14.60)	0.791	NS

*OS-FR analysis restricted to patients with recurrence or progression (n = 82). Bold p values indicate p < 0.05. NS = not significant. Abbreviations: HR, hazard ratio; CI, confidence interval; CRS, chemotherapy response score.*

## Data Availability

Dataset available on request from the authors.
